# Transverse optical torque from the magnetic spin angular momentum

**DOI:** 10.1515/nanoph-2024-0406

**Published:** 2024-10-31

**Authors:** Jiquan Wen, Fengling He, Lv Feng, Wanli Lu, Zhifang Lin, Hongxia Zheng, Huajin Chen

**Affiliations:** School of Automation, 66514 Guangxi University of Science and Technology, Liuzhou, Guangxi 545006, China; School of Materials Science and Physics, China University of Mining and Technology, Xuzhou, Jiangsu 221116, China; State Key Laboratory of Surface Physics and Department of Physics, Fudan University, Shanghai 200433, China; School of Electronic Engineering, 66514 Guangxi University of Science and Technology, Liuzhou, Guangxi 545006, China; Guangxi Key Laboratory of Multidimensional Information Fusion for Intelligent Vehicles, Liuzhou, Guangxi 545006, China; Guangxi Earthmoving Machinery Collaborative Innovation Center, Liuzhou, Guangxi 545006, China

**Keywords:** optical manipulation, transverse optical torque, magnetic spin angular momentum, magnetic response

## Abstract

We report a transverse optical torque exerted on a conventional isotropic spherical particle in a direction perpendicular to that of the illuminating wave propagation. By using full-wave simulations and deriving an analytical expression of the transverse optical torque for particle of arbitrary size, the origin of this transverse optical torque is traced exclusively to the magnetic part of the spin angular momentum, regardless of the size and composition of the illuminated particle. To our surprise, for a non-magnetic dielectric particle, the transverse optical torque is found to originate mainly from the magnetic response of the particle, even when the particle size is much smaller than the illuminating wavelength. This is contrary to the general intuition that the electric response of a non-magnetic dielectric particle dominates its magnetic response in the mechanical effect of light, especially in the Rayleigh limit.

## 1 Introduction

The exchange of linear and angular momenta between light and matter results in the exertion of optical forces and torques on particles [1], [2]. This enables various manipulations of particles such as trapping, transportation, or rotating, and thus offers extensive applications in life sciences, biomedicine, and robotics [3], [4], [5], [6], [7], [8], [9], [10], [11]. Optical force, with the well-known application called optical tweezers, has undergone significant advancements since its initial experimental inception in 1986 [12]. While its counterpart, optical torque, has also captured significant interest in recent decades as a means to control the rotational speed and direction of trapped particles [13], [14], [15], [16], [17], [18]. Typically, optical torques induce particle rotation around a direction parallel to the propagation of light, also called longitudinal optical torques, which are contributed to the longitudinal spin or orbital angular momentum of the incident light beams [19], [20], [21]. Besides the lateral torque along the direction transversely to the spin of illumination [22], in recent years, a concept called transverse optical torque has emerged, enabling the rotation of particles around an axis perpendicular to the direction of light propagation. The phenomena can be observed in various particles, including V-structures [23], birefringent microparticles [5], and core–shell particles [15], [17], owing to the transverse spin angular momentum (SAM). The mechanical manifestation of the transverse SAM occurs in diverse scenarios, such as evanescent waves [24], surface plasmon polaritons [25], focused beams [26], and multiple plane interference fields [27]. In paraxial propagating fields, the separate electric and magnetic contributions of light spin are deemed equivalent due to the intrinsic dual symmetry between electric and magnetic fields [28]. In some previous studies, transverse optical torques on non-magnetic particles have been solely attributed to the transverse electric SAM [17], [21], [29], [30], consequently limiting the measurement of only the electric component of spin through local dynamical characteristics of light. This limitation is believed to arise from the fundamental dual asymmetry of local light–matter interactions which usually have electric characters. Optical torques from the transverse magnetic SAM have ever been noticed in a interference field [27], however, which is limited to dipole particle and exhibits weak strength, unfavourable for practical application.

In this paper, we demonstrate a transverse optical torque stemming from the transverse magnetic SAM for a spherical particle of arbitrary size immersed in an optical field simply composed of two linearly polarized plane waves. We perform precise numerical calculations by employing the full-wave simulation (FWS) method, which combines the generalized Lorenz–Mie theory [31] with the Maxwell stress tensor approach [32]. The consistent spatial distributions of transverse optical torque and the corresponding transverse magnetic SAM of incident optical field strongly indicate that the origin of the transverse optical torque exerted on particles lies in the transverse magnetic SAM. Furthermore, using the rigorous analytical multipole expansion theory, a general relationship is established between the transverse optical torque and the transverse magnetic SAM of light.

In general, for non-magnetic dielectric particles, the excitation of the electrical response is more prominent than the magnetic response, making it a significant contributor to the mechanical effect of light. However, our findings reveal that, even in the case of absorptive dielectric particles, the transverse optical torques induced by magnetic spin are predominantly influenced by the magnetic response of the particles, with only a minor contribution from the electric response. Additionally, for small particles within the Rayleigh approximation regime, the transverse optical torques originate exclusively from the magnetic response. These findings not only enrich the physical origins of transverse optical torque but also provide an efficient method probing the transverse magnetic SAM.

## 2 Formulations and results

**Figure 1:**
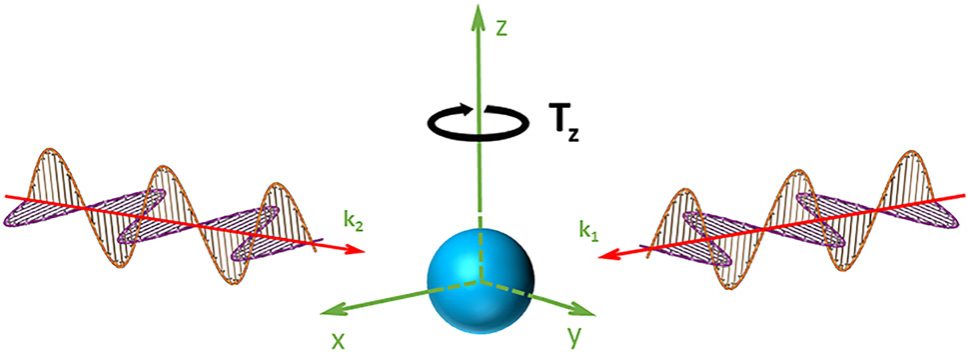
Schematic illustration of a transverse optical torque *T*_*z*_ on a particle in an interference field formed by two linearly polarized plane waves, where *k*_1_ and *k*_2_ denote the wave vectors of the two plane waves, both lying in the *xoy* plane. The background medium is supposed to be vacuum.

As an illustration of this transverse optical torque, we investigate a simple system where a single isotropic spherical particle is illuminated by an interfering optical field composed of two plane waves, as depicted in Figure 1. In this configuration, the wave vectors of both plane waves are confined within the *xoy* plane, and they share the same wavelength *λ* in vacuum. The interfering optical field can be expressed as

(1) $$\begin{array}{cc} E & =E_{1}+E_{2},\\ E_{1} & =\;E_{0}\;E_{1}\;e^{\;i\overset{\cdot }{ı}\;k\;{\hat{k}}_{1}\cdot r},\\ E_{2} & =\;E_{0}\;E_{2}\;e^{\;i\overset{\cdot }{ı}\;k\;{\hat{k}}_{2}\cdot r}, \end{array}$$

where $r=x\hat{x}+y\hat{y}+z\hat{z}$ , *k* = 2*π*/*λ* is the wave number in the background medium. *E*_0_ and $i\overset{\cdot }{ı}$ , respectively, denote the amplitude of the electric field and imaginary unit. The vector ${\hat{k}}_{1}$ and ${\hat{k}}_{2}$ represent the propagation direction of the plane waves with a form

(2) $$\begin{array}{cc} {\hat{k}}_{1} & =\cos\phi_{1}\hat{x}+\sin\phi_{1}\hat{y},\\ {\hat{k}}_{2} & =\cos\phi_{2}\hat{x}+\sin\phi_{2}\hat{y}, \end{array}$$

and the corresponding complex amplitude vectors $E_{1}$ and $E_{2}$ of the plane waves read

(3) $$\begin{array}{cc} E_{1} & =-q\sin\phi_{1}\hat{x}+q\cos\phi_{1}\hat{y}-p\;\hat{z},\\ E_{2} & =-q\sin\phi_{2}\hat{x}+q\cos\phi_{2}\hat{y}-p\;\hat{z}, \end{array}$$

where *p* and *q* are two complex numbers describing different polarized states of wave and satisfy the normalization condition |*p*|^2^ + |*q*|^2^ = 1. The parameters *λ* = 1.064 μm, *E*_0_ = 8.68 × 10^5^ V/m, and linear polarization (*p*, *q*) = (1, 0) are fixed throughout this paper. The time harmonic factor is assumed to be e^−i*ωt*^ and suppressed. The magnetic field **H** can be obtained via Maxwell’s equations. The SAM ⟨**S**⟩ can be split into electric and magnetic contributions as ⟨**S**⟩ = ⟨**S** ^*e*^⟩ + ⟨**S** ^*m*^⟩ with

(4) $$〈S^{e}〉=\frac{\varepsilon_{0}}{4i\overset{\cdot }{ı}\omega }E\times E^{*},\;〈S^{m}〉=\frac{\mu_{0}}{4i\overset{\cdot }{ı}\omega }H\times H^{*},$$

where *ɛ*_0_ and *μ*_0_ are the permittivity and permeability in vacuum while the superscript (^*^) denotes conjugate. The *z* component of SAM $S_{z}^{e}=\left\langle S^{e}\right\rangle \cdot \hat{z}$ and $S_{z}^{m}=\left\langle S^{m}\right\rangle \cdot \hat{z}$ denote, respectively, the transverse electric SAM and transverse magnetic SAM.

**Figure 2:**
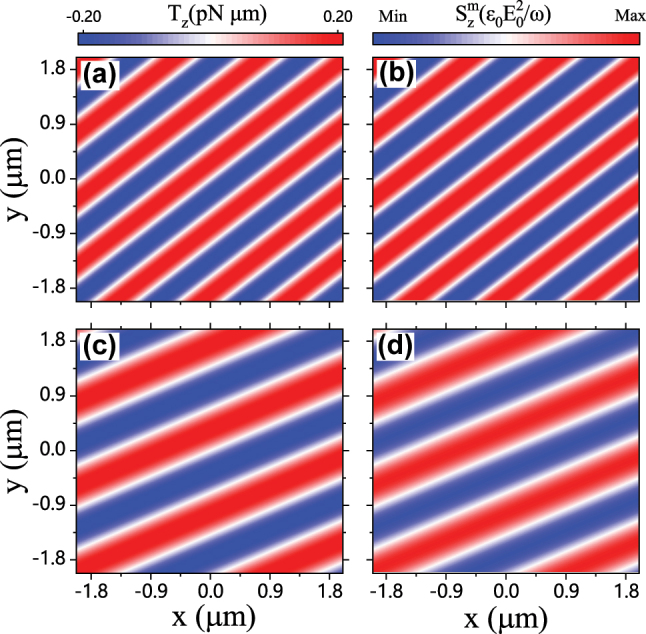
Distributions of (a) transverse optical torque *T*_*z*_ and (b) transverse magnetic SAM $S_{z}^{m}$ vary with particle positions for an Au particle immersed in the field with *ϕ*_1_ = 0° and *ϕ*_2_ = 80°. Profiles of (c) *T*_*z*_ and (d) $S_{z}^{m}$ for a dielectric particle in the field with *ϕ*_1_ = 0° and *ϕ*_2_ = 50°.

Using the FWS method [31], [32], the transverse optical torques *T*_*z*_ of two types of particles (an Au particle and an absorptive dielectric particle) in the optical field given by Eqs. (1)–(3) are numerically calculated as typical examples. Figure 2(a) shows the profile of the transverse optical torque *T*_*z*_ of an Au particle located at different positions of the *xoy* plane, where the particle radius is *r* = 0.4 μm and the permittivity of the Au particle is $\varepsilon_{s}=-50.89+3.54i\overset{\cdot }{ı}$ considering the incident wavelength being *λ* = 1.064 μm, obtained from the Drude dielectric function [33], [34], [35]. The incident angles of the constituent plane waves are respectively set as *ϕ*_1_ = 0° and *ϕ*_2_ = 80°. The distribution of the transverse magnetic SAM $S_{z}^{\text{m}}$ of such an incident beam is presented in Figure 2(b). One can find that spatial distribution of the transverse optical torque *T*_*z*_ in Figure 2(a) and the transverse magnetic SAM $S_{z}^{\text{m}}$ in Figure 2(b) have the exactly same profiles except the difference in magnitude, suggesting that from the light beam perspective, the physical origin of the transverse optical torque may lie in the magnetic spin. This is also supported by the results of absorptive dielectric particle (with *r* = 0.4 μm and $\varepsilon_{s}=2.53+0.1i\overset{\cdot }{ı}$ ) illuminated by optical field with incident angles being *ϕ*_1_ = 0° and *ϕ*_2_ = 50°, as shown in Figure 2(c) and (d). Both typical cases in Figure 2, accompanied with extensive numerical results (data not shown), consistently indicate that the transverse magnetic SAM can be traced as the physical origin of the transverse optical torques exerted on isotropic spherical particles.

We proceed to rigorously investigate the physical mechanism underlying the transverse optical torque using analytical formulas derived within the framework of Cartesian multipole expansion theory [36]. The time-averaged optical torque of a particle in monochromatic optical field is written as [36]

(5) $$\left\langle T\right\rangle =\overset{\infty }{\underset{l=1}{\sum }}\left\langle T_{\text{ext }}^{\left(l\right)}\right\rangle +\overset{\infty }{\underset{l=1}{\sum }}\left\langle T_{\text{rec }}^{\left(l\right)}\right\rangle ,$$

with

(6) ⟨Text (l)⟩=⟨Text e(l)⟩+⟨Text m(l)⟩=∑l=1∞12l!Re(l−1)∇(l−1)Einc *:(l−1)O↔elec (l) −O↔elec (l):(l−1)∇(l−1)Einc *:(2)ϵ↔+∑l=1∞12l!Re(l−1)∇(l−1)Binc *:(l−1) O↔mag(l)−O↔mag (l):(l−1)∇(l−1)Binc *:(2)ϵ↔,⟨Trec (l)⟩=⟨Trec e(l)⟩+⟨Trec m(l)⟩=−18πε0∑l=1∞2l(l+1)k2l+1(2l+1)!ImO↔elec (l):(l−1)O↔elec (l)* +1c2O↔mag(l):(l−1)O↔mag(l)*:(2)ϵ↔,

where the sum runs over the multipoles of order 2^*l*^. The extinction torque $\left\langle T_{\text{ext }}^{\left(l\right)}\right\rangle$ , an analogue of the interception (extinction) optical force, can be understood as the interaction between incident light and oscillating multipoles excited on the particle. The recoil torque $\left\langle T_{\text{rec }}^{\left(l\right)}\right\rangle$ , as the counterpart of the recoil force, stems from the interaction between the oscillating multipoles of the same type (electric or magnetic) and the same order. ***E***_inc_ and ***B***_inc_ are the incident electromagnetic fields, and the superscript symbol ∗ represents a complex conjugate. $\overset{↔}{\epsilon }$ is the Levi-Civita tensor. Fully symmetric traceless *l*-order tensor ${\overset{↔}{O}}_{\text{mag}}^{\left(l\right)}$ and ${\overset{↔}{O}}_{\text{elec}}^{\left(l\right)}$ [36], respectively, denote magnetic and electric multipoles. The order *l* is corresponding to 2^*l*^ pole, such as *l* = 1, 2 denote dipole, quadrupole, respectively. For simplicity, we perform all the derivations in the dimensionless representation by setting *E*_0_ = *B*_0_ = *k* = *ω* = *c* = *ɛ*_0_ = *μ*_0_ = 1, and thus the dimensionless optical torques are in units of $\varepsilon_{0}\;{E_{0}}^{2}$ /*k* ^3^, with *ɛ*_0_ represents the background permittivity.

In the general case, any generic monochromatic optical field in which a particle is immersed is composed of discrete set of homogeneous plane waves

(7) $$\begin{array}{cc} & E=\overset{n_{p}}{\underset{j=1}{\sum }}E_{j}=\overset{n_{p}}{\underset{j=1}{\sum }}E_{j}e^{i\overset{\cdot }{ı}\;k\;{\hat{k}}_{j}\cdot r},\\ & B=\overset{n_{p}}{\underset{j=1}{\sum }}B_{j}=\overset{n_{p}}{\underset{j=1}{\sum }}B_{j}e^{i\overset{\cdot }{ı}\;k\;{\hat{k}}_{j}\cdot r}, \end{array}$$

where *n*_*p*_ is the number of the plane waves making up the optical field. Here, $E_{j}$ and $B_{j}$ are complex amplitude vectors which satisfy

(8) $$\begin{array}{cc} E_{j} & =p_{j}{\hat{\theta }}_{k_{j}}+q_{j}{\hat{\phi }}_{k_{j}},\\ B_{j} & ={\hat{k}}_{j}\times E_{j}, \end{array}$$

where ${\hat{\theta }}_{k_{j}}$ and ${\hat{\phi }}_{k_{j}}$ are the directions of the increasing polar angle and azimuthal angle in the spherical coordinate system for the *j*th wave vector, respectively. The polarization vector *p*_*j*_ and *q*_*j*_ are two complex numbers describing different polarized states of wave and satisfy the normalization condition |*p*_*j*_|^2^ + |*q*_*j*_|^2^ = 1.

Substituting Eqs. (7) and (8) into (5), the general formula for optical torque can be represented by an explicit expression as a product of the Mie coefficients *a*_*l*_, *b*_*l*_ [37], which describe the scattering properties of particles, and the vectors associated with the incident fields, therefore the extinction torque and the recoil torque in Eq. (6) are worked out to be

(9) $$\begin{array}{cc} \left\langle T_{\text{ext }}^{\left(l\right)}\right\rangle & =\left\langle T_{\text{ext }}^{e\left(l\right)}\right\rangle +\left\langle T_{\text{ext }}^{m\left(l\right)}\right\rangle \\ & =\beta_{l}\text{Re}\left(a_{l}\right)\underset{i,j}{\sum }Y_{l,ij}^{\text{exe}}-\beta_{l}\text{Re}\left(b_{l}\right)\underset{i,j}{\sum }Y_{l,ij}^{\text{mxm}},\\ \left\langle T_{\text{rec }}^{\left(l\right)}\right\rangle & =\left\langle T_{\text{rec }}^{e\left(l\right)}\right\rangle +\left\langle T_{\text{rec }}^{m\left(l\right)}\right\rangle \\ & =-\beta_{l}{\left|a_{l}\right|}^{2}\underset{i,j}{\sum }Y_{l,ij}^{\text{exe}}+\beta_{l}{\left|b_{l}\right|}^{2}\underset{i,j}{\sum }Y_{l,ij}^{\text{mxm}}, \end{array}$$

with

$$\beta_{l}=\frac{1}{4}\;\frac{\pi \left(2l+1\right)!}{2^{l}l\left(l+1\right)!\left(2l-1\right)‼}.$$

The **Y** vectors associated with the incident field are given by

(10) $$\begin{array}{cc} \left\langle Y_{l,ij}^{\text{exe}}\right\rangle & =R_{l,ij}^{\left(4\right)}\left[\;Z_{\text{me},ij}^{\left(1\right)}+Z_{\text{me},ij}^{\;\left(1\right)*}\right]+R_{l,ij}^{\left(5\right)}\left[\;Z_{\text{em},ij}^{\left(1\right)*}+Z_{\text{em},ij}^{\left(1\right)}\right]\\ & \;+4i\overset{\cdot }{ı}R_{l,ij}^{\left(7\right)}S_{\text{ee},ij}^{\left(1\right)}+4i\overset{\cdot }{ı}R_{l,ij}^{\left(6\right)}S_{\text{mm},ij}^{\left(1\right)},\\ \left\langle Y_{l,ij}^{\text{mxm}}\right\rangle & =R_{l,ij}^{\left(4\right)}\left[Z_{\text{em},ij}^{\left(1\right)}+Z_{\text{em},ij}^{\left(1\right)*}\right]+R_{l,ij}^{\left(5\right)}\left[Z_{\text{me},ij}^{\left(1\right)*}+Z_{\text{me},ij}^{\left(1\right)}\right]\\ & \;-4i\overset{\cdot }{ı}R_{l,ij}^{\left(7\right)}S_{\text{mm},ij}^{\left(1\right)}-4i\overset{\cdot }{ı}R_{l,ij}^{\left(6\right)}S_{\text{ee},ij}^{\left(1\right)}, \end{array}$$

where *i* and *j* index the plane waves. $Z_{\text{me},ij}^{\left(1\right)}$ , $Z_{\text{em},ij}^{\left(1\right)}$ , $S_{\text{ee},ij}^{\left(1\right)}$ , $S_{\text{mm},ij}^{\left(1\right)}$ are some quantities associated with the incident field, defined by

(11) $$\begin{array}{cc} Z_{\text{me},ij}^{\left(1\right)} & =\frac{1}{2}\;\left[\nabla D_{\text{me},ij}^{\left(1\right)}-\nabla \times S_{\text{me},ij}^{\left(1\right)}-i\overset{\cdot }{ı}\;\left(S_{\text{ee},ij}^{\left(1\right)}+S_{\text{mm},ij}^{\left(1\right)}\right)\right],\\ Z_{\text{em},ij}^{\left(1\right)} & =\frac{1}{2}\;\left[\nabla D_{\text{em},ij}^{\left(1\right)}-\nabla \times S_{\text{em},ij}^{\left(1\right)}+i\overset{\cdot }{ı}\;\left(S_{\text{ee},ij}^{\left(1\right)}+S_{\text{mm},ij}^{\left(1\right)}\right)\right], \end{array}$$

with the field moments for each pair of plane waves

(12) $$\begin{array}{cc} \nabla D_{\text{me},ij}^{\left(1\right)} & =i\overset{\cdot }{ı}\left(k_{i}-k_{j}\right)\;\left(B_{i}\cdot E_{j}^{*}\right)\;e^{i\overset{\cdot }{ı}\;\left(k_{i}-k_{j}\right)\cdot r},\\ \nabla D_{\text{em},ij}^{\left(1\right)} & =i\overset{\cdot }{ı}\left(k_{i}-k_{j}\right)\;\left(E_{i}\cdot B_{j}^{*}\right)\;e^{i\overset{\cdot }{ı}\;\left(k_{i}-k_{j}\right)\cdot r},\\ \nabla \times S_{\text{me},\;ij}^{\left(1\right)} & =i\overset{\cdot }{ı}\left(k_{i}-k_{j}\right)\;\left(B_{i}\times E_{j}^{*}\right)\;e^{i\overset{\cdot }{ı}\;\left(k_{i}-k_{j}\right)\cdot r},\\ \nabla \times S_{\text{em},\;ij}^{\left(1\right)} & =i\overset{\cdot }{ı}\left(k_{i}-k_{j}\right)\;\left(E_{i}\times B_{j}^{*}\right)\;e^{i\overset{\cdot }{ı}\;\left(k_{i}-k_{j}\right)\cdot r},\\ S_{\text{ee},ij}^{\left(1\right)} & =\left(E_{i}\times E_{j}^{*}\right)\;e^{i\overset{\cdot }{ı}\;\left(k_{i}-k_{j}\right)\cdot r},\\ S_{\text{mm},\;ij}^{\left(1\right)} & =\left(B_{i}\times B_{j}^{*}\right)\;e^{i\overset{\cdot }{ı}\;\left(k_{i}-k_{j}\right)\cdot r}. \end{array}$$

The coefficients *R* in Eq. (10) are given by

(13) Rl,ij(4)=∑m=2l(2l+1−m)(2)(2l+1−2m)×[2m2l−m(m+1)(m−2)]Pl−m(xij),Rl,ij(5)=∑m=1l(m+1)(2)(m−1)(2l−m)×(2l+2−m)(2l+1−2m)Pl−m(xij),Rl,ij(6)=∑m=2lm(2)(2l+1−m)(2l+1−2m)Pl−m(xij),Rl,ij(7)=∑m=1l(2l+1−2m)(2)2l2−2(m−1)l+m2−m×Pl−m(xij),

where the summation $\overset{l}{\underset{m=1}{\sum }}$ ^(2)^ and $\overset{l}{\underset{m=1}{\sum }}$ ^(2)^ above represent the index *m* odd and even positive integers satisfying 0 < *m* ≤ *l*, and *P*_*l*_(*x*) is the Legendre polynomial.

In the interfering optical fields composed of two plane waves, given by Eqs. (1)–(3), the *z* components of the field moments, obtained through some algebraic manipulations by applying summation ∑_*i*,*j*_ over the field moments for each pair of plane waves {*i*, *j*} in Eq. (12), can be expressed as

(14) $$\begin{array}{cc} {\left(\nabla D_{me}^{\left(1\right)}\right)}_{z} & ={\left(\nabla D_{em}^{\left(1\right)}\right)}_{z}=S_{\text{ee},\;z}^{\left(1\right)}=0,\\ {\left(\nabla \times S_{me}^{\left(1\right)}\right)}_{z} & =-2\sin\left(\phi_{1}-\phi_{2}\right)\sin\left[x\;\left(\cos\phi_{1}-\cos\phi_{2}\right)\right.\\ & \left.\;+y\;\left(\sin\phi_{1}-\sin\phi_{2}\right)\right],\\ {\left(\nabla \times S_{em}^{\left(1\right)}\right)}_{z} & =2\sin\left(\phi_{1}-\phi_{2}\right)\sin\left[x\;\left(\cos\phi_{1}-\cos\phi_{2}\right)\right.\\ & \left.\;+y\;\left(\sin\phi_{1}-\sin\phi_{2}\right)\right],\\ S_{\text{mm},\;z}^{\left(1\right)} & =-2i\overset{\cdot }{ı}\sin\left(\phi_{1}-\phi_{2}\right)\sin\left[x\;\left(\cos\phi_{1}-\cos\phi_{2}\right)\right.\\ & \left.\;+y\;\left(\sin\phi_{1}-\sin\phi_{2}\right)\right]. \end{array}$$

Notably, in the illuminating optical field described by Eqs. (1)–(3), the electric components of the spin angular momentum density $S_{z}^{e}$ exactly vanishes, since $S_{\text{ee},\;z}^{\left(1\right)}=0$ . Substituting Eq. (14) into (11), one arrives at

(15) $$\begin{array}{cccc} {\left(Z_{me}^{\left(1\right)}\right)}_{z} & ={\left(Z_{em}^{\left(1\right)}\right)}_{z} & = & 0. \end{array}$$

Thus Eq. (9) can be rewritten as

(16) $$\begin{array}{cc} {\left\langle T_{\text{ext }}^{\left(l\right)}\right\rangle }_{z} & ={\left\langle T_{\text{ext }}^{e\left(l\right)}\right\rangle }_{z}+{\left\langle T_{\text{ext }}^{m\left(l\right)}\right\rangle }_{z}\\ & =\beta_{l}\text{Re}\left(a_{l}\right)\;4i\overset{\cdot }{ı}R_{l}^{\left(6\right)}\left(x_{12}\right)S_{\text{mm},\;z}^{\left(1\right)}\\ & \;+\beta_{l}\text{Re}\left(b_{l}\right)\;4i\overset{\cdot }{ı}R_{l}^{\left(7\right)}\left(x_{12}\right)S_{\text{mm},\;z}^{\left(1\right)},\\ {\left\langle T_{\text{rec }}^{\left(l\right)}\right\rangle }_{z} & ={\left\langle T_{\text{rec }}^{e\left(l\right)}\right\rangle }_{z}+{\left\langle T_{\text{rec }}^{m\left(l\right)}\right\rangle }_{z}\\ & =-\beta_{l}{\left|a_{l}\right|}^{2}\;4i\overset{\cdot }{ı}R_{l}^{\left(6\right)}\left(x_{12}\right)S_{\text{mm},\;z}^{\left(1\right)}\\ & \;-\beta_{l}{\left|b_{l}\right|}^{2}\;4i\overset{\cdot }{ı}R_{l}^{\left(7\right)}\left(x_{12}\right)S_{\text{mm},\;z}^{\left(1\right)},\; \end{array}$$

and thus the transverse optical torque can be expressed in terms of $S_{\text{mm},\;z}^{\left(1\right)}={\left(H\times H^{*}\right)}_{z}$ , with $x_{12}={\hat{k}}_{1}\cdot {\hat{k}}_{2}$ . The coefficients $R_{l}^{\left(6\right)}\left(x_{12}\right)$ and $R_{l}^{\left(7\right)}\left(x_{12}\right)$ are

Rl(6)=∑m=2lm(2)(2l+1−m)(2l+1−2m)Pl−m(x12),Rl(7)=∑m=1l(2l+1−2m)(2)2l2−2(m−1)l+m2−m×Pl−m(x12).

Finally, one can obtain an explicit expression for the transverse optical torque of a particle of arbitrary size immersed in an optical field consisting of two identical linearly polarized plane waves which have the wave vectors lying in the *xoy* plane,

(17) $$\begin{array}{cc} T_{z}^{\left(l\right)} & =\beta_{l}\left[\text{Re}\left(a_{l}\right)-{\left|a_{l}\right|}^{2}\right]\;4i\overset{\cdot }{ı}R_{l}^{\left(6\right)}\left(x_{12}\right)S_{\text{mm},\;z}^{\left(1\right)}\\ & \;+\beta_{l}\left[\text{Re}\left(b_{l}\right)-{\left|b_{l}\right|}^{2}\right]\;4i\overset{\cdot }{ı}R_{l}^{\left(7\right)}\left(x_{12}\right)S_{\text{mm},\;z}^{\left(1\right)}. \end{array}$$

The summation of Eq. (17) over all *l* yields the transverse optical torque. Equation (17) clearly shows that the transverse optical torque can be traced to the coupling of multipoles induced on the particle and a quantities associated solely with the incident optical field. For the purpose of facilitating discussion, we arrive at the explicit form of transverse optical torque $T_{z}=\left\langle T\right\rangle \cdot \hat{z}$ in SI units as

(18) $$T_{z}=T_{z}^{a}+T_{z}^{b},$$

with

(19) $$\begin{array}{cc} & T_{\text{z }}^{a}=\frac{16\;\omega }{k^{3}}\overset{\infty }{\underset{l=1}{\sum }}\beta_{l}\left[\text{Re}\left(a_{l}\right)-{\left|a_{l}\right|}^{2}\right]R_{l}^{\left(6\right)}\left(x_{12}\right)S_{z}^{m},\\ & T_{\text{z }}^{b}=\frac{16\;\omega }{k^{3}}\overset{\infty }{\underset{l=1}{\sum }}\beta_{l}\left[\text{Re}\left(b_{l}\right)-{\left|b_{l}\right|}^{2}\right]R_{l}^{\left(7\right)}\left(x_{12}\right)S_{z}^{m}. \end{array}$$

Equations (18) and (19) elegantly establish a definite relationship between the transverse optical torque experienced by manipulated particles and their inherent properties, as well as the characteristics of the operating light field. From the perspective of the illuminating field, Eqs. (18) and (19) indicate that the transverse optical torque can be unequivocally attributed to the transverse magnetic SAM $S_{z}^{m}$ , irrespective of the angle between the two constituent plane waves except 180°, as well as the size and composition of the particle manipulated. From the particle’s perspective, the transverse optical torque *T*_*z*_ can be decomposed into two terms. The first term, $T_{z}^{a}$ , corresponds to the contribution from the electric response of the particle, while the second term, $T_{z}^{b}$ , arises from the magnetic response. Such decomposition is characterized by the electric and magnetic Mie coefficients (*a*_*l*_ and *b*_*l*_), respectively.

**Figure 3:**
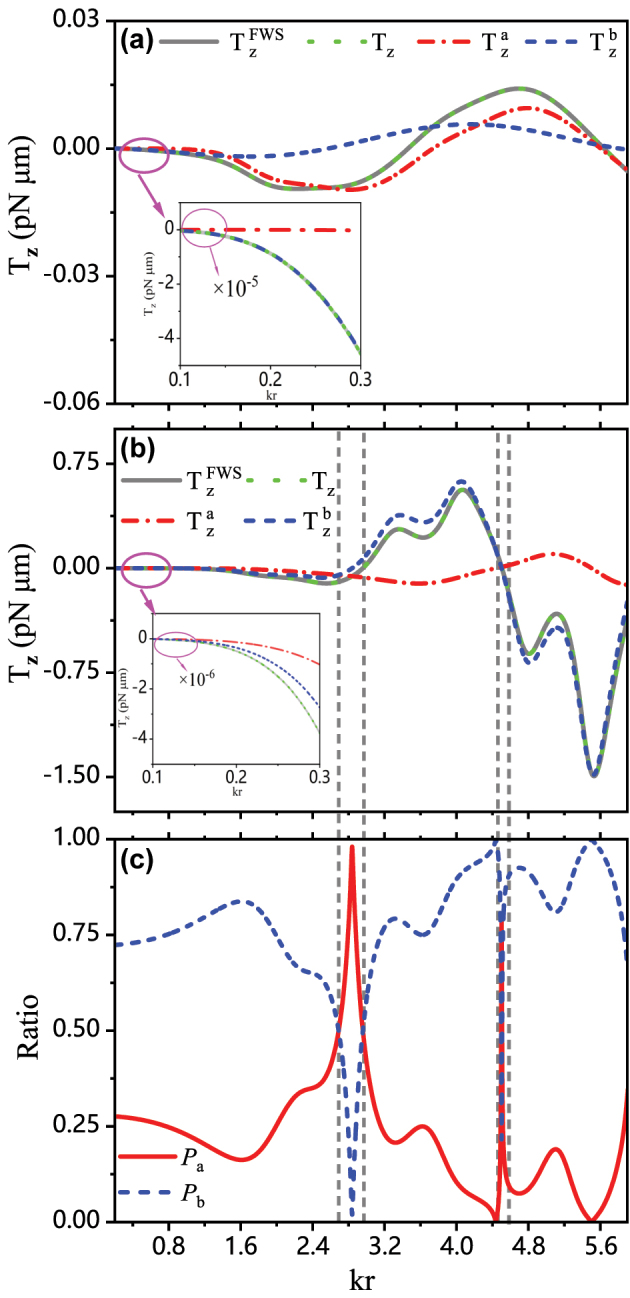
The transverse optical torque $T_{z}^{\text{FWS}}$ using the FWS, *T*_*z*_ using the multipole expansion theory, $T_{z}^{a}$ , and $T_{z}^{b}$ vary with the size parameter *kr* for (a) the Au particle and (b) the dielectric particle. (c) *P*_*a*_(*P*_*b*_) denotes the relative magnitudes of $T_{z}^{a}$ ( $T_{z}^{b}$ ). These particles are immersed in an optical field composed of two plane waves with incident angles *ϕ*_1_ = 0° and *ϕ*_2_ = 80°.

Figure 3 displays the transverse optical torque versus particle size evaluated by the FWS method, contrasted with the results calculated using Eqs. (18) and (19). The remarkable agreement, for both metallic and dielectric particles, corroborates the analytical expressions for the illuminating light fields given by Eqs. (1)–(3). The physical origin of the transverse optical torque presented in our configuration can thus be unambiguously attributed to transverse magnetic SAM $S_{z}^{m}$ . Figure 3 also plots the transverse optical torque contributed by the electric (dash-dotted red lines) and magnetic (dashed blue lines) responses distinguished from the perspective of particles, viz. $T_{z}^{a}$ and $T_{z}^{b}$ presented in Eq. (19), respectively. For the Au particle, as shown in Figure 3(a), one can find that the transverse optical torque arises from the synergistic effect of both the electrical and magnetic responses, with the electrical response being more prominent while the magnetic response also should not be disregarded. For the absorptive dielectric particle shown in Figure 3(b), however, the contribution to the transverse optical torque from the electric response of particle is much smaller than that from the magnetic response, thus the transverse optical torque *T*_*z*_ is primarily determined by the magnetic response $T_{z}^{b}$ , especially in the region where the magnitude of the *T*_*z*_ is notably high. In Figure 3(c), *P*_*a*_ = |*T*_*a*_|/(|*T*_*a*_| + |*T*_*b*_|) and *P*_*b*_ = |*T*_*b*_|/(|*T*_*a*_| + |*T*_*b*_|) are introduced to quantify the relative magnitudes of *T*_*a*_ and *T*_*b*_. It is evident that transverse optical torques arising from magnetic responses significantly outweigh those from electric responses, except in two particular regions (indicated by grey dashed lines) where *T*_*z*_ both approach 0. Specifically, in the range 2.69 < *kr* < 2.96, *T*_*a*_ is predominantly governed by the negative electric quadrupole and electric octupole, leading to *T*_*a*_ < 0 within the whole range. In contrast, *T*_*b*_, determined by negative magnetic dipole and quadrupole as well as positive magnetic octupole, demonstrates a crossover from negative to positive. At *kr* = 4.5, positive and negative components almost cancel each other out, resulting in both nearly negligible *T*_*a*_ and *T*_*b*_ approaching 0 while the relatively large magnitude of *T*_*a*_. This result, as shown in Figure 3(c), challenges our initial intuition regarding the mechanical effect of light on non-magnetic particles, as it unveils a reversal of expectations: the transverse optical torque arising from the magnetic response is unexpectedly prominent, while our inherent impression would suggest a significantly stronger electrical response from the particles. Additionally, we can observe that, for small-sized particles within the Rayleigh dipole-approximation regime, regardless of the particle composition, the electric dipole response induced transverse optical torques $T_{z}^{a}$ vanishes, this is because the factor $R_{l}^{\left(6\right)}$ equals to zero for *l* = 1 when Rayleigh particles are considered. Specifically, the transverse optical torque given by Eqs. (18) and (19) can be reduced as follows

(20) $$\begin{array}{c} T_{z}=T_{z}^{b}=\frac{12\pi \omega }{k_{0}^{3}}\left[\text{Re}\left(b_{1}\right)-{\left|b_{1}\right|}^{2}\right]S_{z}^{m}. \end{array}$$

**Figure 4:**
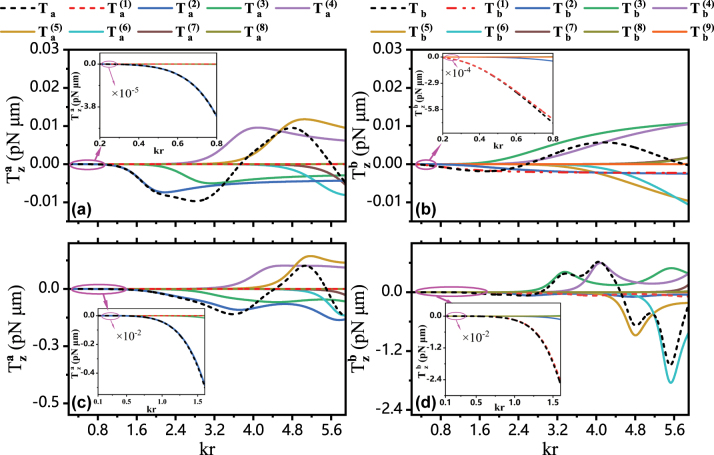
The multipole decomposition of the transverse optical torques $T_{z}^{a}$ and $T_{z}^{b}$ vary with the size parameter *kr* for Au particle in (a), (b) and dielectric particle in (c), (d).

The multipole decomposition of the transverse optical torques is demonstrated in Figure 4 for Au particle and the dielectric particle. It is noted that $T_{a}=\sum_{n=1}^{n_{c}}T_{a}^{\left(n\right)}$ and $T_{b}=\sum_{n=1}^{n_{c}}T_{b}^{\left(n\right)}$ . Here, $T_{a}^{\left(n\right)}$ and $T_{b}^{\left(n\right)}$ correspond to the contributions originating, respectively, the electric response and magnetic response of *n*-order electric and magnetic multipoles, usually referred to as 2^*n*^ poles. The maximum orders *n*_*c*_ at which we terminate the summation are *n*_*c*_ = 8 and *n*_*c*_ = 9 for, respectively, *T*_*a*_ and *T*_*b*_ to guarantee the convergence of the series. The insets of Figure 4(a) and (c) demonstrate that, within the dipole approximation for the smaller *kr* range, *T*_*a*_ for both Au and dielectric particles are entirely determined by $T_{a}^{\left(2\right)}$ since $T_{a}^{\left(1\right)}=0$ , as analyzed based on the Eq. (19) before. However, the *T*_*b*_ is almost determined by $T_{b}^{\left(1\right)}$ while contribution from $T_{b}^{\left(2\right)}$ is negligible for small sized particle, contrast with that of *T*_*a*_, as shown by the insets in Figure 4(b) and (d). As the particle size increases, the higher-order responses from $T_{a}^{\left(3\right)}$ to $T_{a}^{\left(7\right)}$ as well as $T_{b}^{\left(3\right)}$ to $T_{b}^{\left(8\right)}$ are successively excited, contributing significantly to the total *T*_*a*_ and *T*_*b*_.

**Figure 5:**
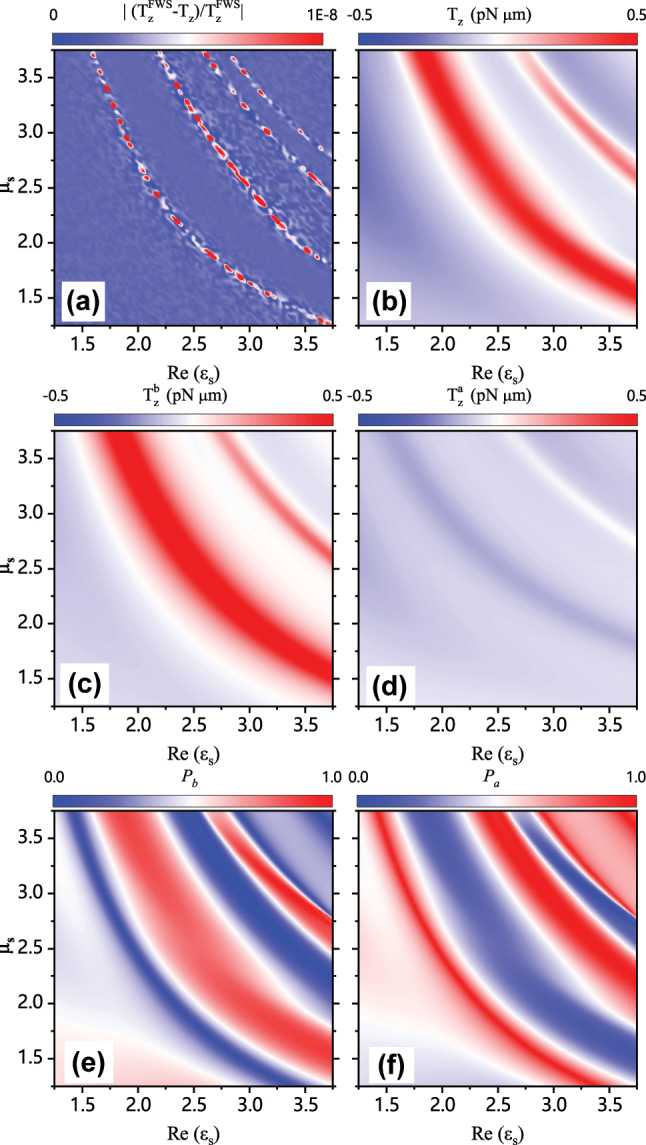
Phase diagrams of (a) the relative discrepancies of *T*_*z*_, (b) *T*_*z*_, (c) $T_{z}^{b}$ , (d) $T_{z}^{a}$ , (e) the relative magnitudes of $T_{z}^{b}$ and (f) the relative magnitudes of $T_{z}^{a}$ , as a function of the permittivity and permeability of the particles. Particles have fixed Im(*ɛ*_*s*_) = 0.1 and radius of *r* = 0.4 μm (*kr* = 2.36), while the two constituent plane waves exhibit incident angles *ϕ*_1_ = 0° and *ϕ*_2_ = 80°.

The remarkable dominance of the magnetic response for absorptive dielectric particles, which governs the transverse optical torques of particles immersed in the interfering field, is not a coincidental discovery limited to the specific particle material presented in Figure 3(b). Figure 5 showcases the transverse optical torque acting on particles across a broad spectrum of material parameters, encompassing varying permittivity and permeability values. The transverse optical torques are calculated using both FWS and analytical expressions in Eqs. (18) and (19), with relative discrepancies $\left|\left(T_{z}^{\text{FWS}}-T_{z}\right)/T_{z}^{\text{FWS}}\right|$ , as depicted in Figure 5(a). It can be observed that the relative discrepancies in various regions, spanning a wide range of particle parameters, approach zero, with values as small as 10^−8^, even in regions where *T*_*z*_ approaches zero. The negligible numerical error observed serves as additional validation for the accuracy and universality of the derived analytical formulations Eqs. (18) and (19), regardless of particle materials and sizes. This reaffirms that the origin of the transverse optical torque can be conclusively attributed to the transverse magnetic SAM, irrespective of particle properties. The transverse optical torque *T*_*z*_, and its decomposed parts $T_{z}^{b}$ as well as $T_{z}^{a}$ , are also plotted in Figure 5(b)–(d), respectively. The contour plots provide a rough demonstration that, for magnetic particles, the contribution of *T*_*z*_ to the overall torque is primarily attributed to $T_{z}^{b}$ , rather than $T_{z}^{a}$ , as discussed earlier in the context of non-magnetic dielectric particles. More quantitatively, the relative magnitude of *T*_*b*_ is shown to be larger than that of *T*_*a*_ in the region where torque strengths are notably high, as evidenced in Figure 5(e) and (f). This confirms the results observed with non-magnetic dielectric particles as shown in Figure 3(b) and (c).

## 3 Conclusions

In summary, we have demonstrated a new type of transverse optical torque that originates from the transverse magnetic SAM of the optical field formed by two plane waves. High-precision FWS method is employed to numerically calculate the optical torques, while the analytical Cartesian multipole expansion method is utilized to uncover the underlying physical mechanism. Within the framework of Cartesian multipole expansion, an explicit expression is established, unveiling the relationship between the transverse optical torque and the transverse magnetic fields spin. This expression holds generally across a wide range of particle parameters, thereby ensuring the broad applicability of the conclusion that the magnetic spin serves as the physical source of the transverse optical torque. Interestingly, for absorptive dielectric particles, the primary contribution to the transverse optical torque arises mostly from the magnetic response of particle, while the contribution from the electric response is negligible. In the case of small-sized dipole Rayleigh particles, regardless of particle properties, the origin of the transverse optical torque can be absolutely attributed to the magnetic response of particle.
